# Impaired Left Atrial Performance Resulting From Age-Related Arial Fibrillation Is Associated With Increased Fibrosis Burden: Insights From a Clinical Study Combining With an *in vivo* Experiment

**DOI:** 10.3389/fcvm.2020.615065

**Published:** 2021-02-03

**Authors:** Kai-bin Lin, Kan-kai Chen, Shuai Li, Ming-qi Cai, Min-jie Yuan, Yan-peng Wang, Xue Zhang, Meng Wei, Mei-Ling Yan, Xin-Xin Ma, Dong-Yan Zheng, Qi-han Wu, Jing-bo Li, Dong Huang

**Affiliations:** ^1^Heart Center, Shanghai Jiaotong University Affiliated Sixth People's Hospital, School of Medicine, Shanghai Jiaotong University, Shanghai, China; ^2^Department of Cardiology, Huadong Hospital, Fudan University, Shanghai, China; ^3^Department of Ultrasound in Medicine, Shanghai Jiao Tong University Affiliated Sixth People's Hospital, Shanghai Institute of Ultrasound in Medicine, School of Medicine, Shanghai Jiaotong University, Shanghai, China; ^4^NHC Key Lab of Reproduction Regulation (Shanghai Institute of Planned Parenthood Research), Fudan University, Shanghai, China

**Keywords:** age, atrial fibrillation, fibrosis, left atrium performance, echocardiography

## Abstract

**Background:** Atrial fibrillation (AF) is increasingly considered an age-related degenerative disease, whose process is associated with the development of impaired left atrial (LA) performance. However, the subtle dynamic changes of LA performance in AF during aging have yet to be fully elucidated. Atrial fibrosis is a key substrate for the development of AF, but the progression of fibrosis during aging and its relationship with LA dysfunction need to be further explored.

**Methods:** A total of 132 control individuals and 117 persistent AF patients were prospectively studied. Subjects were further stratified into three age groups (age group 1: younger than 65 years, age group 2: between 65 and 79 years old, and age group 3: older than 80 years). The two-dimensional speckle tracking imaging was carried out for analyzing the alterations in LA function underlying LA remodeling, whereas electroanatomic mapping was performed to investigate LA fibrosis burden. In animal study, aged mice and young mice served as research subjects. Echocardiography and histological staining were used to assess LA performance and fibrosis burden, respectively.

**Results:** Echocardiography showed progressive increases in LA dimension and LA stiffness index, and progressive decreases in LA global longitudinal strain and LA strain rates with advancing age in both AF and control cohorts, which was more prominent in AF cohort. Electroanatomic mapping showed progressive decrease in mean LA voltage and progressive increases in LA surface area, low-voltage area %, and LA volume with advancing age, whereas more significant alterations were observed in AF patients. Moreover, left atrial global longitudinal strain was positively correlated with mean LA voltage, whereas LA stiffness index was negatively related to mean LA voltage. In animal experiment, increased LA size and pulmonary artery dimension as well as longer P-wave duration and more prominent LA fibrosis were found in aged mice.

**Conclusions:** This study provides new evidence of subtle changes in structure and performance of left atrium and their association with atrial fibrosis in both AF and non-AF subjects during physiological aging. In addition, our study also provides normal values for LA structure and performance in both AF and non-AF conditions during aging. These measurements may provide an early marker for onset of AF and LA adverse remodeling.

## Introduction

Atrial fibrillation (AF) is a common arrhythmia associated with severe adverse clinical outcomes in practice. The incidence of AF ascends sharply with age, casting a heavy burden on health care systems (1). It was predicted that more than 8.3 million patients over the age of 60 will suffer from AF by the year 2030 in China (2). When AF is refractory and sustains with age, afflicted patients tend to develop severe left atrial (LA) dysfunction and heart failure with preserved ejection fraction (3).

Atrial fibrosis is the hallmark of structural remodeling in AF and is considered as the substrate for AF perpetuation (4). Aging contributes to the development of atrial fibrosis, which in turn impairs cardiac function (5). However, an early study illustrated that the degree of atrial fibrosis measured in magnetic resonance imaging was independent of AF duration (6). Furthermore, human autopsy studies indicated aging has a limited contribution to fibrosis development in AF patients (7, 8). When it comes to *in vitro* experiment, it was demonstrated that collagen incubation resulted in shortened action potential duration of pulmonary vein cardiomyocytes (9). However, aging independently prolonged atrial effective refractory period in human (10). Thus, there is a considerable controversy regarding the relationship between AF and fibrosis in the context of aging.

LA strain analysis allows us to quantify subtle cardiac alterations objectively under the background of physiological aging (11–13). Meanwhile, LA strain is applied to appraise the AF burden derived from atrial fibrosis (14). However, integrative analysis of the effect of aging and AF on LA function and their association with atrial fibrosis burden is still limited so far. The aim of the present clinical study was to demonstrate the impact of age-related AF on LA performance and characterize the relationship between LA dysfunction and atrial fibrosis burden. Additionally, animal experiments were also carried out in attempt to elucidating the overall mutual links in deep.

## Methods

### Participants Enrollment

The clinical study was a prospective, single-center study designed to evaluate the alterations of LA function and fibrosis burden in age-related AF patients. It was registered at http://www.chictr.org.cn/index.aspx (ChiCTR-ROC-17011691). The individuals referred to Shanghai Jiaotong University affiliated Shanghai Sixth People's Hospital for catheter ablation between January 2017 and June 2020 were recruited for this study. The control candidates were defined as the patients who received ablation for supraventricular tachycardia with left accessory pathway, severe vasovagal syncope, or lone premature atrial contractions. Persistent AF was defined according to the 2014 American Heart Association/American College of Cardiology/Heart Rhythm Society and 2016 Focused Update of Canadian Cardiovascular Society guidelines for management of the patients with AF (15, 16). Patients with persistent AF undergoing initial catheter ablation were recruited to the AF group. The patients were excluded if diagnosed with or had a previous history of rheumatic heart diseases, cardiac surgery, ST-segment elevated myocardial infarction, and severe heart failure.

All patients were further stratified into three subgroups according to age: age group 1 (<65 years old), age group 2 (between 65 and 79 years old), and age group 3 (>80 years old).

#### Human Echocardiographic Assessment

**Figure 1:**
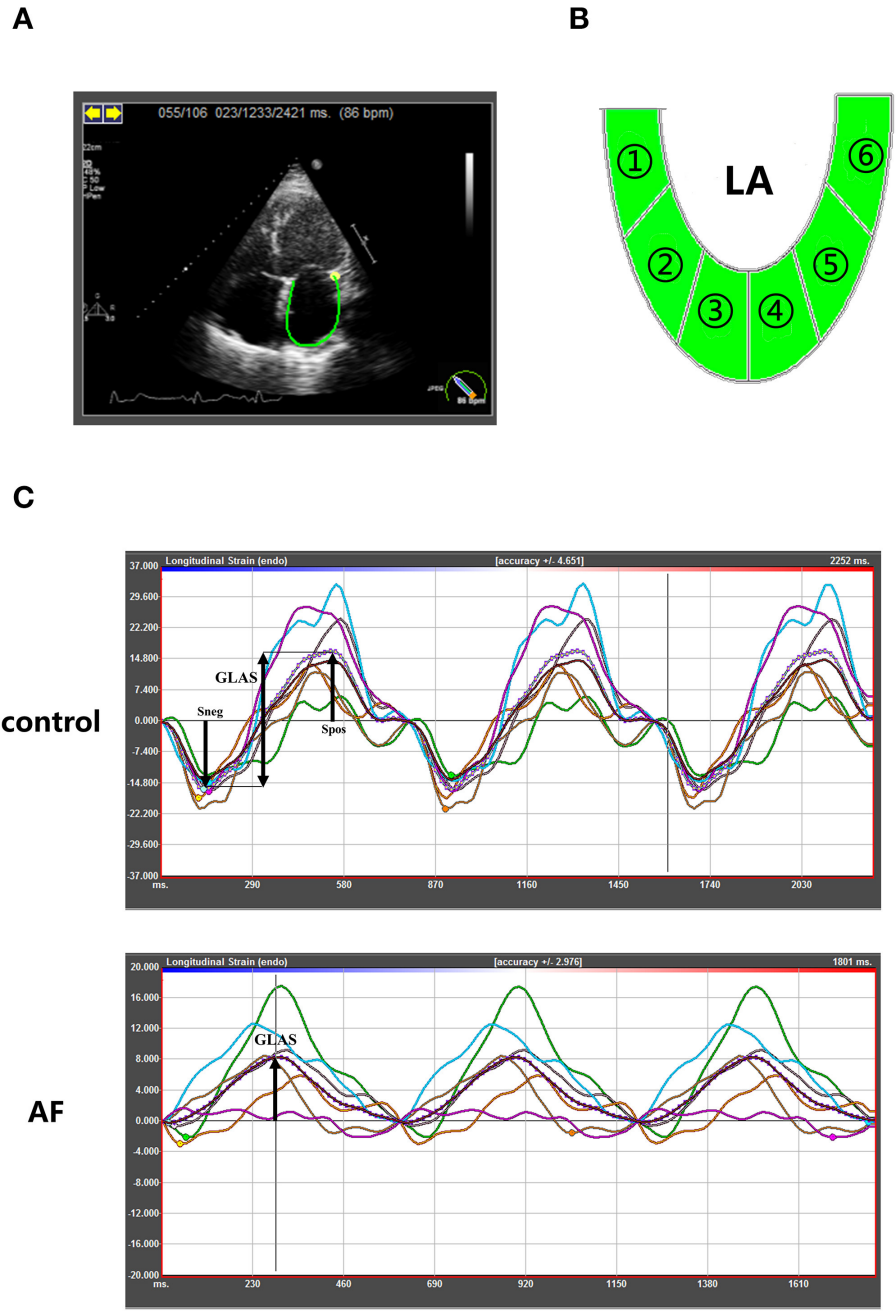
Speckle-tracking echocardiography to quantify LA strain in control and AF patients at three age groups. **(A)** Representative apical four-chamber view echocardiogram of a control adult. Along the inner contour of the LA wall, we define the target region. **(B)** The six segments of the LA wall were into strain analysis. **(C)** The strain curves of six segments and average strain curve (dotted line). The GLAS of control patients were defined Spos minus Sneg. In AF patients, GLAS was identified as the value of Spos. GLAS, left atrial global longitudinal strain; Spos, longitudinal peak early-diastole strain; Sneg, longitudinal peak late-diastole strain.

In order to assess the LA performance with or without AF at different ages, all participants underwent echocardiography examination before ablation. The procedure was conducted as described in our previous study (17). The measurements of two-dimensional and tissue Doppler echocardiographic parameters, including LA dimension (LAD), left ventricular end-diastolic dimension (LVEDD), left ventricular end-diastolic posterior wall thickness, left ventricular septal wall thickness, left ventricular ejection fraction (LVEF), pulmonary artery dimension (PAD), E peak velocity and A peak velocity, were carried out using commercially available iE33 instruments (Philips Medical Systems, Koninklijke, the Netherlands) according to the guideline of the American Society of Echocardiography and the European Association of Cardiovascular Imaging (18–20). Left ventricular mass (LVM) index is calculated according to Devereux formula [LVM index = (LVM)/body surface area]. The measurement of LVM is according to the recommendations (18). Then two-dimensional speckle tracking echocardiography was performed offline by analysis of gray-scale images (four-chamber views) using commercially available software (TomTec Imaging Systems GmbH, Unterschleissheim, Germany). LA endocardial borders were manually traced in four-chamber and two-chamber views (Figure 1A), and LA was divided into six segments subsequently (Figure 1B). The software then generated longitudinal deformation curves and measured the mean values of strain and strain rates. Average of at least three beats was taken in per measurement process. LA strain rates (SRa, SRe, and SRs) and LA global longitudinal strain were analyzed to evaluate LA performance. The LA global longitudinal strain (GLAS) was calculated as the value of longitudinal peak early-diastole strain (Spos, positive peak strain) minus longitudinal peak late-diastole strain (Sneg, negative peak strain). Because of impaired atrial contractility during AF rhythm, Sneg was close to 0. GLAS of AF patients was identified as the value of Spos (Figure 1C). On this basis, LA stiffness index based on strain imaging was defined as the ratio of E/e′ to GLAS [LA stiffness index = (E/e′ ratio)/GLAS] as demonstrated previously (21). All the procedures were performed by two fixed operators.

In this study, we also assessed all parameters' variability in a randomly selected subset of 10 patients referred to our previous study (17). The variability coefficients were all lower than 10% for all measurements in both interobserver and intraobserver analyses.

#### Electroanatomic Mapping

The mapping procedure of all enrolled adults was performed to appraise atrial fibrosis burden. Ensite NAVX system (Abbott, Chicago, IL, USA) was used for the reconstruction and voltage mapping. The mean voltage of LA was defined as the mean value of six-position voltage, including the roof and bottom of LA, anterior LA wall, posterior LA wall, lateral LA wall, and LA appendage. Abbott's 10-pole ring electrodes (81594, Abbott) were used in the measurement process. The fibrosis area was defined as the low-voltage area (LVA) whose endocardial bipolar voltage values were lower than 0.5 mV, observed in at least three adjacent voltage points as previously reported in our study (22). Electrogram quality was assessed by consensus of two experienced observers. A stable contact between the local atrial tissue and the parallel-aligned tip of the ablation catheter was required. The mapping points that displayed poor contact or an unstable electrogram signal were excluded. Quantitative evaluation of LA volume and mean LA voltage were assessed using three-dimensional mapping using standardized software (Abbott). The proportion of LVA in LA surface area (LVA%) was calculated as the formulate (LVA% = $\frac{sum\;of\;LVA}{LA\;surface\;area}*100\%$). All individuals were graded based on the proportion of LVA (LVA%): non-LVA, mild (LVA% ≤10%), moderate (LVA% 10%– <20%), and severe (LVA% ≥20%) as the grading algorithm proposed by Wang et al. (23).

#### Animal Study Protocol

Young mice (3 months old) and senescent mice (27 months old) (C57BL/6, SIPPR-BK Laboratory Animal Co. Ltd., Shanghai, China) were used to evaluate the LA performance by high-frequency ultrasound following inhalation anesthesia of isoflurane (*n* = 15 for each group). Then, surface electrocardiogram (ECG) parameters were measured. Finally, they were sacrificed by cervical dislocation, and organs were immediately harvested.

#### Animal Echocardiographic Assessment

Echocardiography was performed using Vevo 770 imaging system (VisualSonics, Toronto, Ontario, Canada) equipped with a 30-MHz high-frequency scan head. During mild isoflurane anesthesia, the measurement procedure and ordinary echocardiographic parameters refer to a previous work (24). LA size was assessed under the apical four-chamber view. PAD was further measured under parasternal left ventricular long-axis view.

#### ECG Parameter Recording

All mice were anesthetized with 1% sodium pentobarbital at a dose of 0.1 mg/g. Surface limb leads were recorded continuously during the study through electrodes. ECG was shown and filtered (0.5–250 Hz) with a multiple electroconductive physiological recorder (Hongtong, Shanghai, China). ECG recordings at baseline were made at 100–400 mm/s paper speed. RR interval, P-wave duration, PR interval, and QT interval were calculated in lead II.

#### Fibrosis Area Measurement

Masson trichrome-stained sections were carried out to examine cardiac collagen accumulation. Collagen area fraction was determined as previously reported (25).

### Statistical Analysis

Continuous variables were expressed as mean ± standard deviation for normally distributed data. Other continuous variables were presented as median (25th−75th percentile). Statistical significance of differences between groups was assessed using Student *t*-test for data or analysis of variance with normal distribution. Differences between quantitative non-normal variables were studied by non-parametric tests (Kruskal–Wallis *H*-test). All categorical variables were presented as frequencies and percentage, and the χ^2^-test or Fisher exact test was used, as appropriate. Mantel-Haenszel χ^2^-test was used to measure the correlation between two ordinal categorical variables. Pearson correlation was computed to evaluate the associations between two continuous variables. Stepwise multiple regression analysis was performed to evaluate the presence of collinearity for factors and determine the predictors for target continuous variables. Statistical analyses were performed using SPSS, version 17.0.2 (SPSS, Inc., Chicago, IL, USA). All *P* values were two-sided, and *P* < 0.05 was considered statistically significant.

## Results

### Participants' Characteristics

A total of 303 candidates were enrolled. Fifty-four subjects were excluded because of poor image quality. The study cohort included 132 control individuals (supraventricular tachycardia with left accessory pathway for ablation: 42 cases; severe vasovagal syncope for catheter ablation of ganglionated plexi: 18 cases; premature atrial contractions for ablation: 72 cases) and 117 persistent AF patients, respectively. The clinical characteristics of the patients are presented in Table 1. In the AF group, AF duration prolonged with advanced age (age group 2 vs. age group 1, age group 3 vs. age group 2, all *P* < 0.05). There was no significant difference in the gender and BMI between control and AF patients across age-matched subgroups. There were more patients in age group 3 who also suffered from hypertension or diabetes mellitus, as compared with age group 1, with or without the presence of AF (all *P* < 0.05). In the AF group, the elderly patients (age group 2 and age group 3) had higher CHA_2_DS_2_-VASc scores than that in the young patients (age group 1, all *P* < 0.05). When it came to the HAS-BLED score, a graded increase was observed among different age groups (0.6 ± 0.1 vs. 1.8 ± 0.7 vs. 2.5 ± 1.0, all *P* < 0.05). The exposure to inhibitors of angiotensin-converting enzyme inhibitor/angiotensin receptor blocker, statins, and β-blocker therapy did not differ in the control and AF cohorts or among different age groups.

**Table 1:** Baseline characteristics for all subgroups of patients.

	**Control (*****n*** **= 132)**			**AF patients (*****n*** **= 117)**		
	**Age group 1**	**Age group 2**	**Age group 3**	**Age group 1**	**Age group 2**	**Age group 3**
	***n* = 67**	***n* = 43**	***n* = 22**	***n* = 27**	***n* = 48**	***n* = 42**
Age (year)	48.8 ± 7.5	72.8 ± 4.4	81.9 ± 2.1	54.8 ± 10.3	73.7 ± 5.3	82.1 ± 3.1
Male (%)	40 (59.3%)	24 (55.8%)	13 (59.1%)	17 (62.9%)	28 (58.3%)	24 (57.1%)
Time since diagnosis (month)	NA	NA	NA	8.5 (1–31)	13 (2–44)	18.5 (2–63)
BMI (kg/m^2^)	21.7 ± 3.8	20.1 ± 4.9	18.3 ± 6.6	21.8 ± 4.2	20.4 ± 6.9	18.1+4.2
Hypertension, *n* (%)	20 (29.8%)	20 (46.5%)	12 (54.5%)	9 (33.3%)	29 (60.4%)	30 (71.4%)
DM, *n* (%)	8 (11.9%)	13 (30.2%)	7 (31.8%)	6 (22.2%)	10 (25.8%)	23 (54.8%)
CHA_2_DS_2_-VASc score	NA	NA	NA	1 (0-3)	2 (1–4)	3 (2–5)
HAS-BLED score	NA	NA	NA	0.6 ± 0.1	1.8 ± 0.7	2.5 ± 1.0
ACEI/ARB, *n* (%)	17 (25.4%)	18 (41.9%)	9 (40.9%)	8 (29.6%)	25 (52.1%)	26 (61.9%)
β-Blocker, *n* (%)	35 (52.2%)	26 (60.4%)	14 (63.6%)	12 (44.4%)	27 (56.3%)	26 (61.9%)
Statins, *n* (%)	27 (40.3%)	28 (65.1%)	15 (68.2%)	16 (59.2%)	34 (70.8%)	30 (71.4%)

*AF, atrial fibrillation; BMI, body mass index; HF, heart failure; EF, ejection fraction; DM, diabetes mellitus; ACEI, angiotensin-converting enzyme inhibitor; ARB, angiotensin receptor blocker; NA, not applicable*.

### LA Dysfunction During Aging and the Effect of AF on LA Function

**Figure 2:**
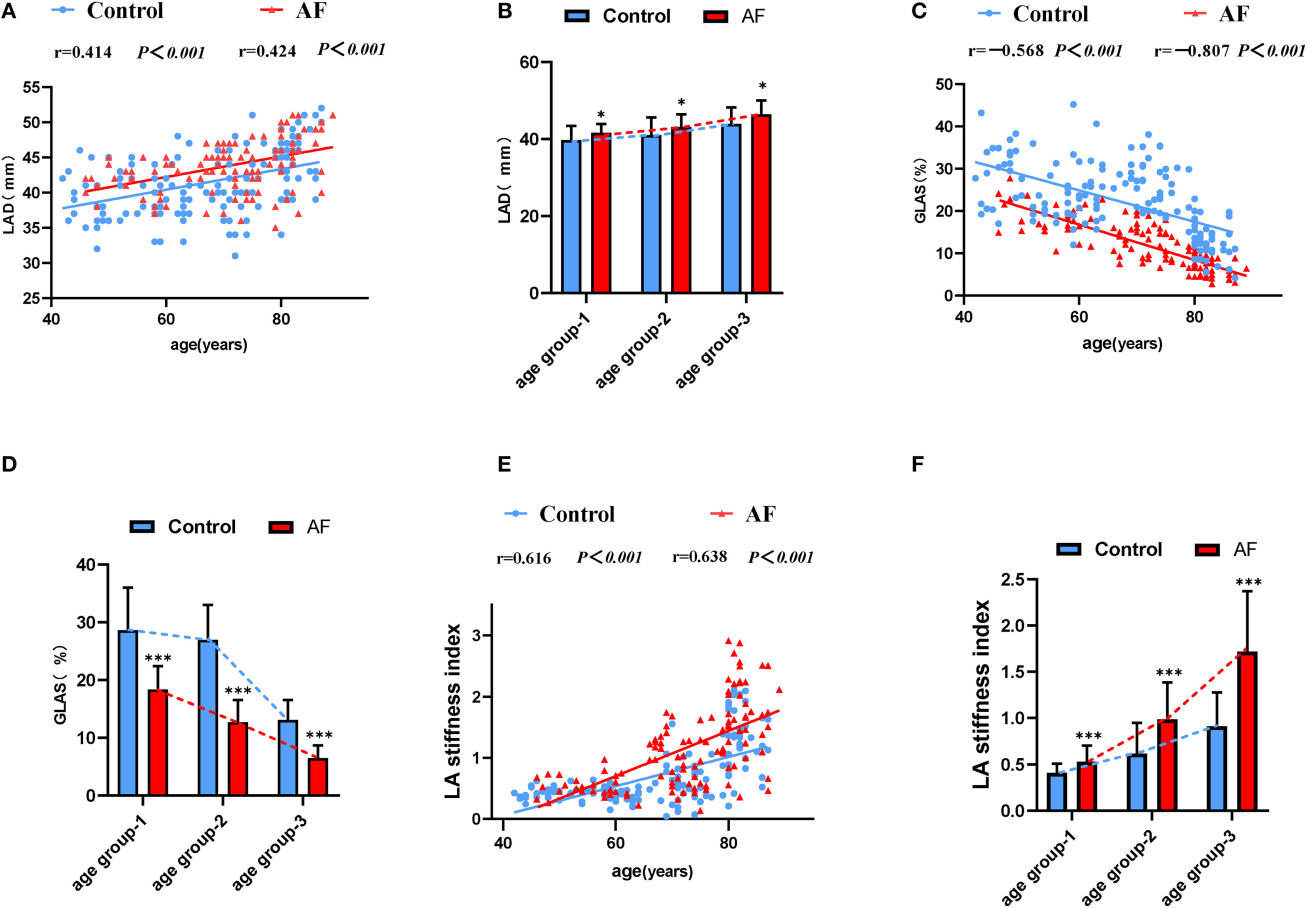
The LA performance in the control individuals and AF patients during physiological aging. **(A)** Correlations of LAD and age in control adults and AF patients. **(B)** The effect of AF on LAD during aging. **(C)** Correlations of GLAS and age in control adults and AF patients. **(D)** The worse GLAS was shown in AF patients during aging. **(E)** A strong positive association between LA stiffness index and age. **(F)** Changes for LA stiffness index in the absence and presence of AF during aging. LAD, left atrial dimension; GLAS, left atrial global longitudinal strain. *P < 0.05 vs. control in age-match groups; **P < 0.01 vs. control in age-match groups; ***P < 0.001 vs. control in age-match groups.

Table 2 summarizes echocardiographic characteristics of control individuals and AF patients in different subgroups. In three age-matched groups, similar LVEF was presented in the control individuals and AF patients. Meanwhile, in both control and AF cohorts, LVEDD, LV mass index, and E velocity remained stable along with age. In the control cohort, the elderly adults (age groups 2 and 3) showed higher A velocity in the comparison with young adults (age group 1), whereas the E/A ratio did not differ across different age groups (*P* > 0.05). PAD and E/e′ ratio increased with age in both control and AF cohorts (age group 3 vs. age group 1, *P* < 0.05, respectively). Besides, AF patients had a significantly higher E/e′ ratio than control (all *P* < 0.05) in elderly patients (age groups 2 and 3), whereas the difference in PAD was observed only in age group 2. LAD, representing LA size, would help to identify abnormal atrial substrate and estimate the arrhythmia burden (26). A positive correlation between LAD and age was observed (control: *r* = 0.414; AF: *r* = 0.424, all *P* < 0.001; Figure 2A) Meanwhile, LAD was greater in AF patients compared to the control adults of corresponding age group (all *P* < 0.05; Figure 2B). GLAS was found to be negatively correlated with age in both control and AF cohorts (control: *r* = −0.568; AF: *r* = −0.807, all *P* < 0.001; Figure 2C), which was more significant in AF cohort (all *P* < 0.001; Figure 2D). Besides, stepwise multiple regression analysis showed that considering the covariates of gender, hypertension, and diabetes mellitus, age (β = −0.37, *P* < 0.001) and AF status (β = −8.13, *P* < 0.001) were both independently correlated with the GLAS. The LA stiffness index is a novel measure to estimate LA compliance and function (27). In the present study, LA stiffness exhibits a positive correlation with age in both cohorts (control: *r* = 0.616; AF: *r* = 0.638, all *P* < 0.001; Figure 2E), with the AF cohort showing a higher value than its age-matched control (all *P* < 0.001; Figure 2F). As for LA strain rates (SRa, SRs, and SRe), SRa and SRe were negatively correlated with age in both control individuals and AF subjects (SRa, control: *r* = −0.381, AF: *r* = −0.335, all *P* < 0.001; SRe, control: *r* = −0.289, AF: *r* = −0.248, all *P* < 0.01), whereas such correlation was not detected for SRs. It is worth noticing that in patients older than 80 years (age group 3), SRa, SRs, and SRe were all significantly lower in the AF cohort as compared with control cohort.

**Table 2:** The echocardiographic characteristics for all age groups of patients.

		**Control (*****n*** **= 132)**			**AF patients (*****n*** **= 117)**			***P*-value in control individuals**	***P-*value in AF patients**
		**Age group 1**	**Age group 2**	**Age group 3**	**Age group 1**	**Age group 2**	**Age group 3**		
		***n* = 67**	***n* = 43**	***n* = 22**	***n* = 27**	***n* = 48**	***n* = 42**		
LVEF (%)		55.7 ± 6.8	55.9 ± 7.9	55.0 ± 11.8	54.8 ± 6.2	54.6 ± 8.7	53.9 ± 10.3	0.165	0.104
LVEDD (mm)		41.7 ± 2.6	42.4 ± 4.8	42.2 ± 3.5	42.3 ± 1.6	42.5 ± 2.9	42.7 ± 4.6	0.505	0.663
LVM index (g/m^2^)		79.8 ± 14.8	80.4 ± 19.7	81.2 ± 18.4	80.9 ± 13.9	80.8 ± 20.7	81.0 ± 25.6	0.150	0.204
E velocity, m/s		0.80 ± 0.29	0.73 ± 0.25	0.73 ± 0.27	0.82 ± 0.22	0.79 ± 0.24^†^	0.75 ± 0.29^†^	0.247	0.115
A velocity, m/s		0.61 ± 0.14	0.67 ± 0.20	0.70 ± 0.21	NA	NA	NA	0.060	NA
E/A ratio		1.2 ± 0.4	1.1 ± 0.3	1.0 ± 0.4	NA	NA	NA	0.286	NA
E/e′ ratio		9.6 ± 1.6	9.5 ± 2.3	10.1 ± 2.6	9.8 ± 2.0	10.4 ± 3.9^†^	10.9 ± 4.0^‡^	0.074	0.051
PAD (mm)		22.4 ± 2.3	22.1 ± 2.7	23.9 ± 2.8	24.1 ± 3.6	24.2 ± 2.9^†^	24.9 ± 3.0	0.055	0.087
LAD (mm)		39.7 ± 3.6	41.3 ± 4.6	43.1 ± 5.2	41.7 ± 3.3^*^	43.1 ± 4.2^†^	46.4 ± 3.7^‡^	<0.001	<0.001
GLAS (%)		25.7 ± 7.2	23.2 ± 8.1	12.4 ± 3.6	18.4 ± 4.0^***^	12.7 ± 3.8^†††^	6.8 ± 2.1^‡‡^	<0.001	<0.001
LA stiffness index		0.4 ± 0.1	0.6 ± 0.3	0.9 ± 0.4	0.6 ± 0.2^***^	0.9 ± 0.4^†††^	1.6 ± 0.8^‡‡‡^	<0.001	<0.001
LA strain rate	SRa	−3.1 ± 0.8	−2.8 ± 0.8	−2.5 ± 0.7	−2.2 ± 0.6^***^	−2.1 ± 0.6^†††^	−1.4 ± 0.5^‡‡‡^	<0.001	<0.001
	SRs	2.6 ± 0.8	2.3 ± 0.7	1.9 ± 0.6	2.2 ± 0.6^*^	1.9 ± 0.6^†^	1.7 ± 0.4	<0.001	<0.001
	SRe	−2.0 ± 0.5	−1.4 ± 0.5	−1.0 ± 0.4	−1.5 ± 0.5^*^	−0.9 ± 0.3^†††^	−0.8 ± 0.3^‡^	<0.001	<0.001

*AF, atrial fibrillation; LAD, left atrial dimension; LVEF, left ventricular ejection fraction; LVEDD, left ventricular end-diastolic dimension; GLAS, left atrial global longitudinal strain; SRs, global longitudinal left atrial stain rate; SRe, global longitudinal early–diastolic left atrial stain rate; SRa, global longitudinal late–diastolic left atrial stain rate; PAD, pulmonary artery dimension*.

* P < 0.05 vs. control in age group 1;

*** P < 0.001 vs. control in age group 1;

† P < 0.05 vs. control in age group 2;

††† P < 0.001 vs. control in age group 2;

‡ P < 0.05 vs. control in age group 3;

‡‡ P < 0.01 vs. control in age group 3;

‡‡‡ *P < 0.001 vs. control in age group 3. NA, not applicable*.

### Evolution of Electrical Characteristics of LA in Patients With AF During Aging

**Figure 3:**
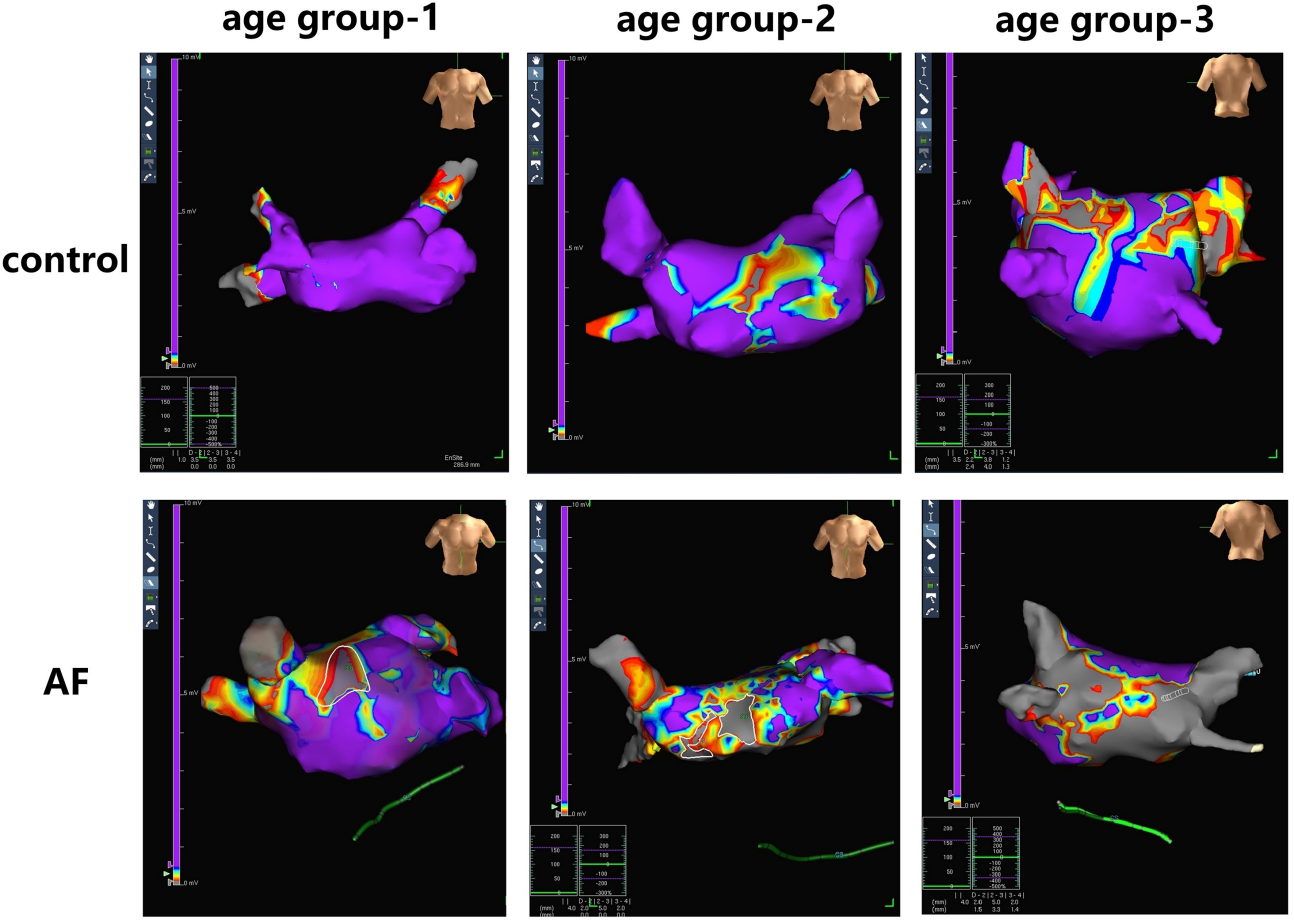
Examples of LA fibrosis burden in control individuals and AF patients at three age groups. Different values of electrical voltage representing LA fibrosis are color-coded in purple to gray. Purple region, gray region and other color region represent completely healthy myocardial tissue with a voltage ≥0.5 mV, scar with a voltage ≤0.1 mV, and junction area at the voltage of 0.1- to 0.5-mV region, respectively.

**Figure 4:**
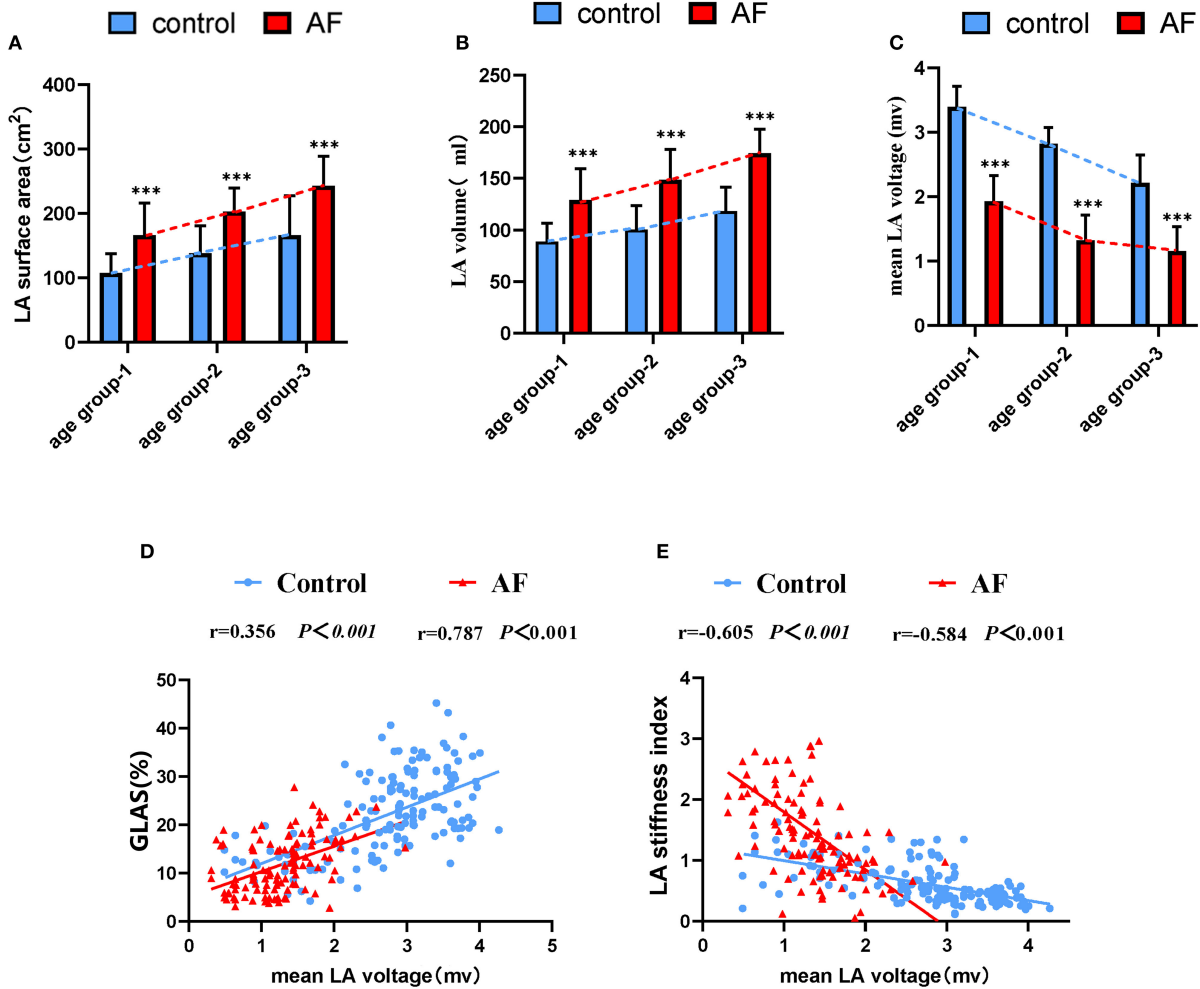
The relationship between the LA dysfunction and fibrosis progression under the background of atrial aging. **(A,B)** In addition to increased LA surface area and LA volume significantly correlating with advanced age, alterations in two measurements were more apparent in AF patients in aging process. **(C)** Changes for mean LA voltage in the absence and presence of AF during aging. **(D,E)** Correlations of GLAS and LA stiffness index with mean LA voltage in control adults and AF patients. LA, left atrium; AF, atrial fibrillation; GLAS, left atrial global longitudinal strain. *P < 0.05 vs. control in age-match groups; **P < 0.01 vs. control in age-match groups; ***P < 0.001 vs. control in age-match groups.

LA electroanatomic mapping was performed to further confirm whether the marked alterations of LA performance were associated with atrial fibrosis in age-related AF patients (Table 3). Figure 3 shows representative evolution patterns of LVA in control individuals and AF patients under the process of physiological aging. LA surface area and LA volume showed proportional increments, along with age, in both AF and control cohorts. Comparing with control subjects, AF patients showed greater LA volume and LA surface area than control subjects in corresponding age groups (Figures 4A,B). Mean LA voltage decreased with age in both cohorts, whereas it was lower in AF patients than in control subjects in corresponding age group (Figure 4C). In addition, stepwise multiple regression analysis illustrated that age (β = −0.04, *t* = −16.33, *P* < 0.001) and AF status (β = −1.26, *t* = −19.61, *P* < 0.001) were independently associated with mean LA voltage after adjustment for other factors (gender, hypertension, and diabetes mellitus). LVA% grades increased with age, irrespective of AF status (control: *r* = 0.369, AF: *r* = 0.392, all *P* < 0.001). Besides, in the comparison with age-matched control individuals, AF patients tended to have higher LVA% grades, although we only observed a significant difference in LVA% distribution in age groups 1 and 2 (all *P* < 0.05). Moreover, there was a significant positive linear relation between GLAS and mean LA voltage (control: *r* = 0.356, *P* < 0.001; AF: *r* = 0.787, *P* < 0.001; Figure 4D), whereas a negative correlation was found between LA stiffness index and mean LA voltage (control: *r* = −0.605, *P* < 0.001; AF: *r* = −0.584, *P* < 0.001; Figure 4E). Further adjustments for gender, hypertension, and diabetes mellitus did not materially alter these results.

**Table 3:** The mapping results for all age groups of control and AF patient.

	**Control (*****n*** **= 132)**			**AF patients (*****n*** **= 117)**			***P*-value for control individuals**	***P*-value for AF patients**
	**≤65y**	**66–80y**	**>80y**	**≤65y**	**66–80y**	**>80y**		
	***n* = 67**	***n* = 43**	***n* = 22**	***n* = 27**	***n* = 48**	***n* = 42**		
LA surface area (cm^2^)	107.1 ± 30.9	146.3 ± 30.9	152.4 ± 63.2	178.4 ± 50.2^***^	210.6 ± 47.5^†††^	240.6 ± 63.0^‡‡‡^	<0.001	<0.001
LA volume (cm^3^)	88.6 ± 28.0	100.6 ± 36.0	118.1 ± 43.5	129.0 ± 42.20^***^	148.7 ± 59.6^†††^	164.6 ± 63.0^‡‡‡^	<0.001	0.008
Mean LA voltage (mV)	3.4 ± 0.4	2.8 ± 0.3	1.6 ± 0.7	1.9 ± 0.4^***^	1.2 ± 0.4^†††^	1.0 ± 0.4^‡‡‡^	<0.001	<0.001
LVA% grouping, *n* (%)								
Non-LVA	63 (94.0%)	36 (83.7%)	13 (59.1%)	17 (62.9%)^***^	20 (41.6%)^†††^	8 (19.0%)	<0.001	0.005
Mild LVA	4 (6.0%)	5 (11.6%)	5 (22.7%)	6 (22.2%)	14 (29.17%)	11 (26.2%)		
Moderate LVA	0 (0%)	2 (4.6%)	2 (9.1%)	2 (3.7%)	10 (20.8%)	8 (19.0%)		
Severe LVA	0 (0%)	0 (0%)	2 (9.1%)	2 (3.7%)	4 (8.3%)	15 (35.7%)		

*P < 0.05 vs. control in age group 1;

**P < 0.01 vs. control in age group 2;

*** P < 0.001 vs. control in age group 1;

† P < 0.05 vs. control in age group 2;

†† P < 0.01 vs. control in age group 2;

††† P < 0.001 vs. control in age group 2;

‡ P < 0.05 vs. control in age group 3;

‡‡ P < 0.01 vs. control in age group 3;

‡‡‡ *P < 0.001 vs. control in age group 3*.

### An Evaluation for LA Remodeling Using High-Frequency Ultrasound and the Association With Atrial Fibrosis in Aged Mice

A preclinically experiment was also performed to investigate the relationship between age-related LA remodeling and atrial fibrosis burden. Table 4 shows the echocardiographic and Doppler measurements of cardiac function in the young (3-month-old) and aged (27-month-old) male C57BL/6 mice. Interventricular septal thickness, left ventricular internal-diastolic dimension, and left ventricular end-diastolic volume were all significantly increased in aged mice as compared with young mice, indicating the presence of age-related cardiac remodeling. Despite the lack of statistical significance, a marginally increase in E/e′ ratio was observed in aged mice, suggesting a possible presence of age-related diastolic dysfunction.

**Table 4:** Non-invasive measurements of C57BL/6 male mice aged at 3 and 27 months under mild anesthesia.

	**Young (*n* = 15)**	**Aged (*n* = 15)**	***P*-value**
HR (bpm)	411.8 ± 17.80	421.2 ± 29.30	0.100
IVS;d (mm)	0.88 ± 0.08	1.00 ± 0.09	0.056
IVS;s (mm)	1.28 ± 0.12	1.56 ± 0.25	0.060
LVID;d (mm)	4.03 ± 0.17	4.33 ± 0.18	0.030
LVID;s (mm)	2.78 ± 0.27	2.81 ± 0.11	0.790
LVPW;s (mm)	0.75 ± 0.13	0.70 ± 0.10	0.570
LVPW;d (mm)	1.03 ± 0.11	1.07 ± 0.09	0.680
E velocity (m/s)	0.70 ± 0.05	0.67 ± 0.11	0.392
A velocity (m/s)	0.48 ± 0.08	0.38 ± 0.08	0.035
E/A ratio	1.58 ± 0.37	1.50 ± 0.27	0.341
E/e′ ratio	18.66 ± 4.89	24.40 ± 7.00	0.054
FS (%)	31.17 ± 4.68	34.90 ± 2.69	0.054
LVEDV (uL)	71.72 ± 6.19	84.75 ± 8.87	0.030
LVESV (uL)	29.44 ± 7.10	30.07 ± 2.85	0.786

*FS, fractional shortening; HR, heart rate; IVS;d, interventricular septal thickness at diastole; IVS;s, interventricular septal thickness at systole; LV, left ventricular; LVID;d, LV internal -diastolic dimension; LVID;s, LV internal-systolic dimension; LVPW;s, left ventricular posterior wall thickness at end systole; LVPW; d, left ventricular posterior wall thickness at end diastole; E/A, E–A peak velocity ratio; LVEDV, LV end-diastolic volume; LVESV, LV end-systolic volume; LVESD, LV end-systolic dimension; LVESV, LV end-systolic volume*.

**Figure 5:**
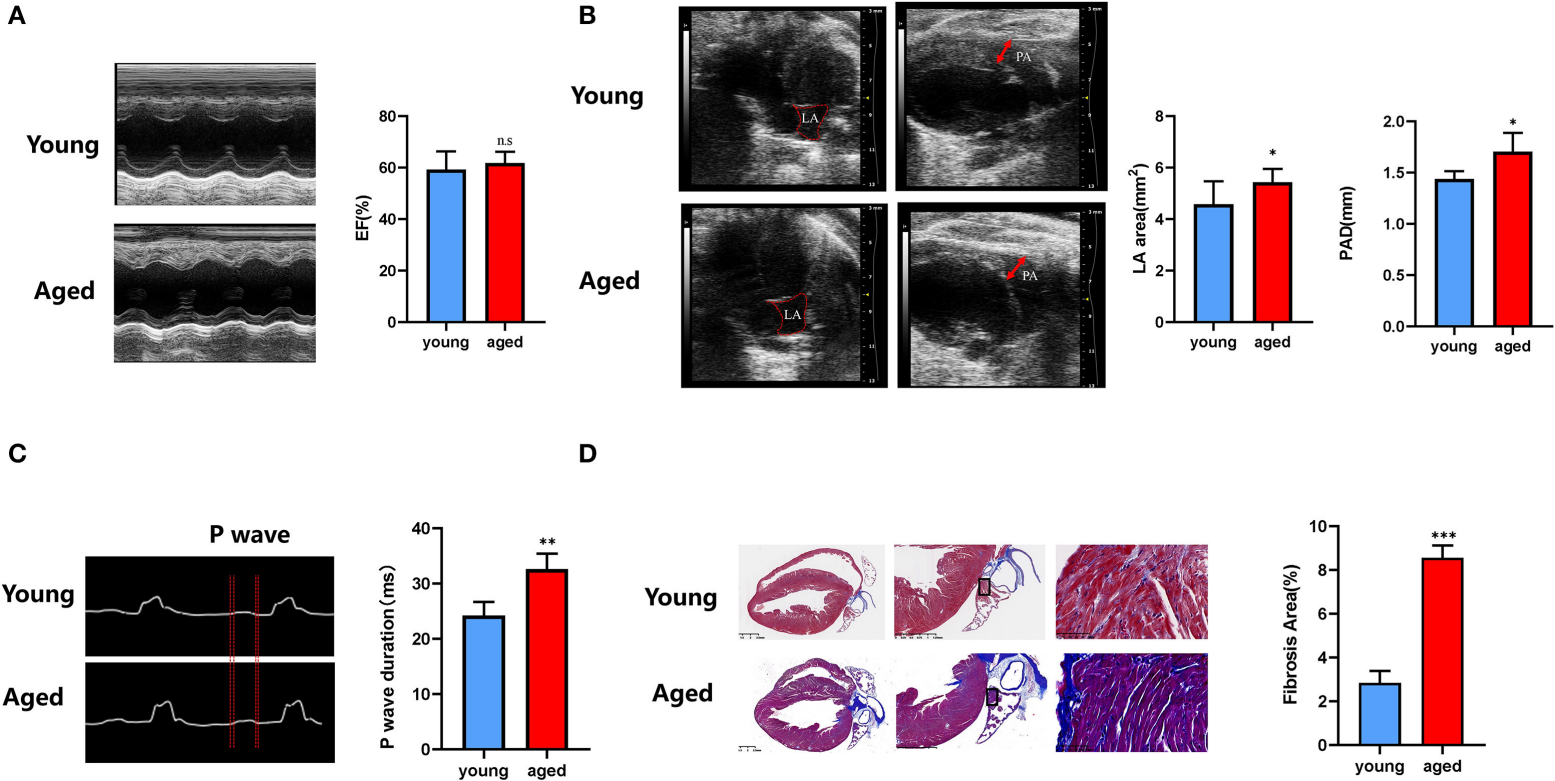
Age-related LA remodeling is associated with atrial fibrosis in mice. **(A)** Representative echocardiographic images of ejection fraction analysis. **(B)** Representative B-mode echocardiographic recordings of LA area and PA diameter and quantification of LA area and PA diameter (right). **(C)** Representative electrocardiographic (ECG) recordings (left) in multiple electroconductive physiological recorder and quantification of P-wave duration. **(D)** Representative results of Masson staining of the left atrium sections obtained from young and old mice, and statistical results of fibrosis area in the left atrium. *n* = 15 in each group. n.s., no significant difference. *P < 0.05, **P < 0.01, ***P < 0.001.

In accordance with the clinical study, LVEF did not differ between young and old mice (59.16 ± 6.71 vs. 61.73 ± 4.16%, *P* = 0.34; Figure 5A), indicating the age-related cardiac alterations conformed to pathophysiologic progression of heart failure with preserved ejection fraction. Non-invasive LA volume and PA diameter were reported to be useful and state-independent measures of age-related cardiac diastolic dysfunction in mice (28). Representative B-mode images of LA and pulmonary artery in young and aged mice are shown in Figure 5B. LA area (4.57 ± 0.85 vs. 5.75 ± 0.75 mm^2^, *P* = 0.01) and PAD (1.44 ± 0.07 vs. 1.71 ± 0.16 mm, *P* = 0.02) were both increased significantly in aged mice. PR interval did not differ between two groups (20.56 ± 2.16 vs 19.50 ± 2.89 ms, *P* > 0.05). Comparing with young mice, aged mice presented shorter RR interval (153.33 ± 9.01 vs 130.67 ± 8.00 ms, *P* < 0.001), along with longer P-wave duration (24.17 ± 2.27 vs. 32.60 ± 2.49 ms, *P* < 0.01; Figure 5C), indicating the increased inducibility of AF (29, 30). Moreover, in Masson trichrome stain, larger fibrosis areas were observed in aged mice (Figure 5D), suggesting aggravation of LA fibrosis during aging.

## Discussion

Impaired LA performance is increasingly considered as a predictor for aging and AF (31–33). Although a few circulating molecules were reported to be correlated with age-related cardiovascular disease progress, none of them is capable of accurately manifesting cardiac performance in real time (34). Therefore, the present study aimed to evaluate subtle changes in LA performance due to AF during the process of aging. To our knowledge, this is the first report of evaluating subtle changes in mechanical and electrical characteristics of LA in AF patients and comparing them with non-AF control subjects during the process of aging. We found a negative correlation between LA performance and age, which was more prominent with the presence of AF. On that basis, we also illustrated through electroanatomic mapping that the alterations in LA performance were associated with development of LA fibrosis. Finally, in a murine model, we further validated the changes in LA performance and the relationship between impaired LA performance and fibrosis during aging.

In the present study, LAD tested by echocardiology, LA surface area, and LA volume tested by electroanatomic mapping all increased with age in both AF and control cohorts, with AF patients displaying a greater LA size evidenced by all three parameters compared with control individuals at corresponding age, indicating the LA remodeling progression in both AF and non-AF subjects during aging. However, a prior study reported aging does not significantly alter indexed LA size until the age of 80 years in normal healthy subjects (35). We suspected this discrepancy may have something to do with the control cohort of the present study, which were patients who received ablation for other arrhythmia instead of healthy subjects. Previous studies showed that LA strain allows early detection of age-related LA dysfunction underlying LA remodeling (36). In current study, we demonstrated the age-related LA abnormalities in both control and AF patients *via* evaluating the dynamic changes in GLAS and LA strain rates, in accordance with work from other groups (37). Of note, in elderly patients with higher prevalence of hypertension and diabetes mellitus, LA remodeling and consequent LA dysfunction were more severe. Meanwhile, decreased GLAS and LA strain rates were observed in the AF patients comparing with the age-matched control individuals. LA stiffness index, measured in an invasive method, is considered as a novel index to assess LA dysfunction (15, 27, 38). The current study measured the LA stiffness index in a non-invasive manner based on strain imaging and systematically described its dynamic changes in AF and control patients in the context of aging. Similar to the results of invasive assessment (27), we observed that high LA stiffness index was associated with advanced age and the presence of AF. These findings illustrated the nature of consecutive progression of cardiac aging and the effect of AF on the longitudinal progression.

The relationship between fibrosis burden and LA performance in AF and non-AF subjects during aging was also evaluated in our study. The results of voltage mapping showed a graded decrease in mean LA voltage and a graded increase in LVA% grade alone with age, which is in accordance with an early observation in a large cross-sectional sample with AF (39). Of note, to our knowledge, this is the first study in which electroanatomic mapping results were acquired from non-AF subjects of different age groups. The results help to reveal the evolution pattern of LA electrical characteristics during aging. Moreover, in the current study, AF patients displayed lower LA voltage and higher LVA% grade as compared with non-AF subjects at corresponding age, except for subjects older than 80 years (age group 3), in which LVA% grades in AF cohort were not significantly different from those of control cohort. We deduce that the effect of aging might overrun the effect of AF in subjects older than 80 years. It is worth mentioning that we had excluded some measurements because of inadequate contact between the catheter and atrial wall, as was reported in a previous study (40). LA stiffness index has been proven to be related to LVA in AF patients (27, 40). LVA is affected by multiple factors, such as the size and shape of the heart. As a matter of fact, mean LA voltage appears to be a better way to describe overall LA interstitial fibrosis level (41, 42). In the present study, we discovered a significant positive link between the mean LA voltage and GLAS, and a significant negative link between the mean LA voltage and LA stiffness index. These findings provide further evidence to support the idea that aging enhances susceptibility of AF *via* promoting the progression of LA fibrosis.

Corresponding to our clinical findings, in the animal study, a prolonged P-wave duration was shown in aged mice, which provided further evidence for increased susceptibility of AF in aged subjects (43). A significant age-dependent increase of LAD was reported in a murine model with Acuson CV-70 (Siemens) (44). In the current study, aged mice showed expanded LA size (representing by LA area), in accordance with our clinical study. In the present study, increased PAD was observed in aged mice, which is consistent with the clinical findings. We also performed Masson trichrome stain to illustrate histological changes of LA during aging, the result showed a significant increase in LA fibrosis burden in aged mice, which was consistent with the electroanatomic mapping results in our clinical study.

There are also some limitations to the present study that could be addressed in the future. First, this is a single-center study with limited sample size, and the control cohort comprised patients who received ablation for other arrhythmias instead of healthy subjects, which may result in selection bias. Second, the accurate measurement of LA performance in a murine model can be challenging. The use of three-dimensional echocardiograph could potentially improve the sensitivity and accuracy of the tests. Lastly, in order to evaluate dynamic changes in LA performance during aging, a longitudinal study may be more convincing, especially when sample size is limited.

## Conclusion

This study provides new evidences of subtle changes in mechanical and electrical characteristics of LA during process of aging in both AF and non-AF subjects. There was a negative correlation between LA performance and age, and the presence of AF further impairs LA performance in an additive manner, which is probably ascribed to increased atrial fibrosis burden, as evidenced by results from both electroanatomic mapping in human study and histological staining in animal experiment. In addition, our study also provides normal values for LA mechanical and electrical characteristics in both AF and non-AF conditions during aging. These measurements may provide an early marker for the onset of AF.

## Data Availability Statement

The original contributions presented in the study are included in the article/supplementary material, further inquiries can be directed to the corresponding authors.

## Ethics Statement

The studies involving human participants were reviewed and approved by Ethics Committee of Shanghai Sixth People's Hospital. The patients/participants provided their written informed consent to participate in this study. The animal study was reviewed and approved by Institutional Animal Care and Use Committee (IACUC) of Shanghai Sixth People's Hospital. Written informed consent was obtained from the individual(s) for the publication of any potentially identifiable images or data included in this article.

## Author Contributions

K-bL, K-kC, and SL performed the clinical study, experiments, and drafted the original manuscript. M-jY, M-qC, M-LY, and XZ enrolled the patients and analysis part of clinical data. X-XM collected the ultrasound imaging and guided the cardiologists to analysis the data. D-YZ, MW, Y-pW, and Q-hW read critically and revised the manuscript. DH and J-bL reviewed and edited the manuscript. All authors contributed to the article and approved the submitted version.

## Conflict of Interest

The authors declare that the research was conducted in the absence of any commercial or financial relationships that could be construed as a potential conflict of interest.
